# Personalized prevention and population health impact: how can public health professionals be more engaged?

**DOI:** 10.1093/eurpub/ckaa018

**Published:** 2020-07-20

**Authors:** Stefania Boccia, Walter Ricciardi

**Affiliations:** c1 Sezione di Igiene, Istituto di Sanità Pubblica, Università Cattolica del Sacro Cuore, Roma, Italia; c2 Department of Woman and Child Health and Public Health - Public Health Area, Fondazione Policlinico Universitario A. Gemelli IRCCS, Roma, Italia

Since 2000 incredible advancement in genotyping technology, coupled with the reduction in the costs of genome sequencing and the more recent advent of digital technologies in healthcare including wearable devices and mHealth, initiated a third revolution in medicine. Such technologies are creating unprecedented opportunities for disease prevention, diagnosis and treatment and for disease monitoring on a personalized basis, both within the health system and beyond. As all the new technologies and their applications advance, they are likely to play an integral role in shaping healthcare and the ways in which citizens manage and optimize their health.^1^

While we acknowledge that preventive activities in healthcare represent a key pillar for guarantee health system sustainability, such innovative in diseases’ prevention are not yet fulfilled. As highlighted by the European Steering Group on Sustainable Healthcare and more recently reaffirmed in the State of the Health of EU of the Companion report^2^, the implementation of sustainable healthcare requires a shift from treatment of established disease, to disease prevention and early diagnosis, and it relies on the need to engage citizens to take greater responsibility for their health. In fact, despite the tremendous increase in life expectancy, the latest Eurostat data reports that the average number of years of life lived with some disability in the EU is around 18. Given the potential for effective preventive efforts in postponing the onset of disabilities and reducing healthcare costs, the expectation is that the current ‘one size fit all’ approaches in prevention take advantage of the new technologies in healthcare in order to be targeted at those who need more. The expectations to realize such personalized approach in preventive healthcare are not new. Already in 2008 an editorial reported that ‘if preventive care could be provided only to those who are going to get the illness, it would be more effective and cost-effective’.^3^ More than 10 years later, however, we are still struggling to collect high quality evidences on the efficacy, effectiveness and efficiency of personalized approaches in prevention, ideally in the context of broad Health Technology Assessment evaluations.^4^ Additionally, we need to engage public health professionals. In 2018, some scientists argued that whereas public health starts with populations, the word ‘precision’ or personalized implies a concern only with individuals.^5^ Although a later editorial from Lancet replied that ‘precision public health … should not be feared. It should be embraced’,^6^ the more traditional public health professionals are still stocked with prejudices that are grounded to some extent. In fact, while Big Data in healthcare already demonstrated their power in providing accurate information for decision makers to target more precise interventions at populations in the greatest need, when we talk about incorporating individual -omic profiling in preventive strategies, the situation is still uncertain. In theory, the -omic profiling might be considered a useful component of health management since birth, but the extent to which it really represents an added value in terms of improved outcomes and quality of life needs additional evidences. Let us consider the polygenic risk score (PRS), which is a number based on variation in multiple genetic loci and their associated weights that can be used to predict a certain diseases’ risk. The use of PRS in health subjects is an important area of development for public health and warrants close attention, but there is still a good deal to learn about how to maximize benefits for population health. In principle, estimating the individual susceptibility of a common adult-onset conditions, is central to clinical decision-making, however, risk prediction does not necessarily implies effective prevention and improved outcomes. Although evidences are accumulating from large retrospective cohort studies across Europe on the ability of PRS to accurately stratify population in subgroups that can differentially benefit from target primary and secondary preventive interventions based on drugs or lifestyles, we still need large prospective studies that demonstrate and quantify the impact of PRS at population level in terms of disease prevention.

Last but not least, all the stakeholders should be engaged in the discussion to properly implement precision public health. We need all professionals in healthcare being literate on the potential and challenges of the use of current technologies in healthcare, and we need an increased health literacy at the population level. Premature translation of innovations in prevention can do more harm than benefit, if people are convinced that extensive self-monitoring with devices is useful, or undergoing a Direct to consumer Genetic test will save their lives. In this sense the advocacy of informed public health professionals is a key issue. In conclusion, scientific innovation offers amazing opportunity: it is our responsibility as scientists to support policy makers in dissecting the hypes from the real value-based interventions. It is not easy in such a complex scenario, but it is even more relevant for those working in public health.

## Funding

The PRECeDI project has received funding from the European Union's Horizon 2020 research and innovation programme MSCA-RISE-2014: Marie Skłodowska-Curie Research and Innovation Staff Exchange (RISE) under the grant agreement No. 645740.

*Conflicts of interest*: None declared.
